# Rebound Thymic Hyperplasia after Recovery from Ectopic Cushing Syndrome: A Case Report and Literature Review

**DOI:** 10.2174/0118715303432270251209104836

**Published:** 2026-04-29

**Authors:** Antonio Musolino, Elisa Cairoli, Alessandro Palleschi, Paola Toja, Manuela Chiodaroli, Sabrina Corbetta, Luca Persani, Valentina Morelli

**Affiliations:** 1 Department of Medical Biotechnology and Translational Medicine, University of Milan, Milan, Italy;; 2 SC Endocrinology-Diabetology Dietetics and Clinical Nutrition, ASST Lariana, Sant'Anna Hospital, San Fermo della Battaglia (CO), Como, Italy;; 3 Department of Pathophysiology and Transplantation, University of Milan, Milan, Italy;; 4 S.C. Thoracic Surgery and Lung Transplantation, Fondazione IRCCS Ca' Granda - Ospedale Maggiore Policlinico, Milan, Italy;; 5 Department of Endocrine and Metabolic Diseases, IRCCS Istituto Auxologico Italiano, Milan, Italy;; 6 Department of Biomedical, Surgical and Dental Sciences, University of Milan, Milan, Italy

**Keywords:** Case reports, cushing syndrome, differential diagnosis, magnetic resonance imaging, neuroendocrine tumors, thymus hyperplasia

## Abstract

**Introduction:**

Rebound thymic hyperplasia (RTH) is a rare benign condition resulting from compensatory proliferation of thymocytes after remission from stressful conditions, including hypercortisolism. Its appearance can sometimes be misleading, making differential diagnosis with thymic epithelial or neuroendocrine tumor challenging.

**Case Presentation:**

We describe the case of a 31-year-old patient who was referred to our outpatient clinic for worsening hirsutism, acne, and fatigue. Biochemical and dynamic tests showed an ACTH-dependent hypercortisolism of ectopic origin (Ectopic Cushing syndrome, ECS). An enhanced total body CT scan was performed to localize the tumor, revealing a 14 mm peribronchial nodule in the left upper lung that was surgically removed, leading to hypercortisolism resolution and hypoadrenalism. During a follow-up chest-abdomen CT scan, an anterior mediastinal enlargement was found. To better characterize the lesion, the patient underwent an enhanced MRI, which provided a conclusive diagnosis of RTH. This allowed the medical team to avoid unnecessary and invasive surgical thymectomy.

**Conclusion:**

The present case highlights the diagnostic importance of recognizing RTH as a benign mimic of thymic tumors in adults recovering from ectopic Cushing syndrome. Misinterpretation may lead to unnecessary surgical intervention. MRI may be useful to characterize RTH in a non-invasive way. Successful treatment requires collaboration among endocrinologists, surgeons, and radiologists.

## 1 INTRODUCTION

Cushing syndrome (CS) is a rare disease characterized by an increased cortisol secretion leading to typical clinical signs and symptoms, such as easy bruising, buffalo hump, central obesity, proximal muscular atrophy, facial plethora, striae rubrae, and mood disorders. Hypercortisolism is also associated with the presence of several comorbidities, of which the most frequent are hypertension, cardio-cerebrovascular events, thromboembolic events, osteoporosis and fragility fractures, diabetes mellitus, and increased mortality risk [1, 2]. In most cases, CS is caused by an ACTH-secreting pituitary adenoma (50-70%, also known as “Cushing disease”), followed by cortisol-producing adrenocortical adenomas (15-25%) and ACTH-secreting ectopic neuroendocrine tumors (10-20%) [3]. Rebound thymic hyperplasia (RTH) is a rare benign condition resulting from compensatory proliferation of thymocytes after remission from stressful conditions, including hypercortisolism. RTH is defined as an increase in size, weight, or volume of the gland at the expense of thymic parenchyma [4]. RTH is a benign condition that is frequent in pediatric age but rare among adults. Physiological thymic adipose involution after the age of 40 explains its rarity, although residual thymic tissue can persist in older adults [5, 6]. Its appearance can sometimes be misleading, making the differential diagnosis with a thymic epithelial or neuroendocrine tumor challenging. Advanced CT and MRI techniques could play a role in its differential diagnosis [7, 8]. We, herein, present the case of a 31-year-old female patient in whom RTH occurred 8 months after recovery from ectopic Cushing syndrome (ECS). Fig. (**1**) shows the patient’s history timeline.

## 2 CASE DESCRIPTION

A 31-year-old female patient referred to our outpatient clinic in August 2023 for worsening hirsutism, acne, fatigue, and 5-kilogram body weight gain over 3 months. Her clinical history was unremarkable except for polycystic ovary syndrome, which was diagnosed at age 15. She was taking estrogen-progestin pill therapy since puberty for mild hirsutism. The physical examination showed signs of grade 1 obesity and mild facial hirsutism: blood pressure was 130/90 mmHg, heart rate was 80 bpm, body weight was 84 kg, and BMI was 30.5 kg/m^2^. Ferriman-Gallway score was 3. The patient practiced regular physical activity, but in the last months, she experienced lower limb myalgia, forcing her to stop exercising. Her medical history was negative for endocrine disease.

Biochemical and hormonal blood tests were performed, and the results are reported in Table (**1**). Increased androgen and high morning cortisol values were found and confirmed 1 month after the estrogen-progestin therapy withdrawal. The biochemical data also revealed high ACTH levels, neutrophilic leukocytosis, hypokalemia, and urinary free cortisol (UFC) x13 the upper limit of normality.

Suspecting an ACTH-dependent Cushing syndrome, CRH-test, desmopressin test, and high-dose dexamethasone suppression tests were performed. We also confirmed high UFC and late-night salivary cortisol (LNSC) (Table **2**). Except for the high dexamethasone doses suppression test, all the findings and clinical presentation were oriented towards an ectopic Cushing syndrome (ECS).

Considering the rapid onset of hypercortisolism, the patient underwent an enhanced total body CT scan to localize the responsible lesion. A 14 mm peri-bronchial nodule was detected in the dorsal segment of the left upper pulmonary lobe (Fig. **2**).

The patient underwent a typical lung segmentectomy (culmen) with uniportal video-assisted technique (VATS) in November 2023; the postoperative course was favorable. Histological analysis showed an atypical well-differentiated (G2) bronchial carcinoid with a mitotic index 4/mm^2^, Ki67 5%, without signs of pleural or nodal invasion, no tumor necrosis, and disease-free resection margins (pT1b, N0). After surgical removal, rapid onset of hypoadrenalism, normalization of potassium levels, and androgen suppression were observed (Table **3**).

The patient started glucocorticoid replacement therapy with cortisone acetate and continued regular biochemical and radiological follow-up. A staging 18FDG PET-CT was performed in February 2024, and areas of pathological hypercaptation were not documented. In July 2024, an enhanced chest and abdominal CT scan was performed. An anterior mediastinal enlargement of 17x30 mm was detected; the lesion appeared hyperdense (65 HU; Fig. **3a**). Regular biochemical and hormonal assessments were conducted, and an ECS recurrence was excluded. To rule out a thymic primitive neoplasm or a thymic metastasis, the patient underwent an enhanced MR scan soon afterward. As shown in Figs. (**3b**-**3d**), the lesion appeared dimensionally stable and hyperintense on T2-weighted images; it was characterized by decreased signal intensity in out-of-phase images without signal restriction on diffusion-weighted images, leading to the diagnosis of RTH. Radiological diagnosis allowed avoiding diagnostic thymectomy.

## 3 DISCUSSION

RTH is a benign condition; it is frequent in pediatric age, but is considered rare among adults. Physiological thymic adipose involution after the age of 40 supports this interpretation, although small amounts of residual thymic tissue can still be found in older adults [9, 10]. Two types of thymic hyperplasia are recognized: thymic lymphoid follicular hyperplasia and true thymic hyperplasia. In lymphoid follicular hyperplasia, the thymus is characterized by enlarged lymphoid follicles, and it is frequently observed in patients with myasthenia gravis and other autoimmune disorders, including Graves’ disease and Sjogren syndrome [11-13]. The thymus is not enlarged in this form of hyperplasia; the term refers to the hyperplastic appearance of lymphoid follicles. True thymic hyperplasia, on the other hand, is defined as an increase in size, weight, or volume of the gland and is not associated with lymphoid follicular hyperplasia. The diagnosis of true thymic hyperplasia can often be difficult to make, and it can be confused with thymoma or other tumors [4, 14]. RTH after hypercortisolism remission is a rare cause of true thymic hyperplasia; the postulated pathophysiological mechanism is compensatory hyperplasia after glucocorticoid-induced atrophy interruption. In fact, it has been observed that glucocorticoids can induce an important thymic hypotrophy, and they were also historically used to reduce thymus gland size and make cardiac shadow more evident for diagnostic purposes in children [9]. For this reason, glucocorticoids have been studied as a possible therapy for thymomas [15, 16]. Moreover, an independent effect of ACTH on thymocyte growth has been documented; it has been demonstrated that melanocortin receptor type 2 is expressed on thymocytes, and animal models treated with recombinant ACTH showed an increase in thymus gland cellularity [17]. In the setting of recovery from endogenous hypercortisolism, RTH has been reported in 19 cases in the literature. The patients’ mean age at hypercortisolism diagnosis has been reported to be 36 years, ranging from 5 months to 59 years. RTH occurred mostly after 6-8 months from recovery, and spontaneous RTH radiological remission was documented from 11 to 38 months after hypercortisolism remission (Table **4**) [9, 18-25].

In the past decades, histological confirmation was usually necessary to rule out thymic neoplasm, and many reported cases underwent a complete diagnostic thymectomy, which showed benign thymic tissue with diffuse thymic hyperplasia and absence of adrenocorticotropic hormone expression at immunostaining. Thanks to new imaging techniques that can help in the differential diagnosis, it is possible to reduce the need for surgical intervention for this benign condition. Concerning the evaluation of mediastinal masses, CT scan is considered equal or superior to MR, but the latter can better characterize cystic or necrotic components of neoplasms. Typically, RTH presents as a smooth triangular-shaped organ enlargement of 3-5 cm, while thymic epithelial neoplasms appear as large, bulky, and non-homogeneous masses. In some cases, radiological features can overlap, making differential diagnosis more challenging [7]. In this case, MR can be useful thanks to chemical shift imaging, a specific sequence that can outline the presence of intravoxel fat accumulation [26]. Chemical shift imaging relies on the difference in resonance frequency between protons in water and triglyceride molecules. A decrease in signal intensity in out-of-phase sequences indicates microscopic fat accumulation that cannot be detected by CT or other MR fat-suppression sequences. This can be useful to differentiate RTH from thymic neoplasms, where there is no intralesional fat accumulation [27]. In our case, we were able to localize the primary tumor, but this is not always possible in ECS. Occult ECS accounts for 10-20% cases of ECS, and it is defined as the presence of ACTH-dependent hypercortisolism of ectopic origin with negative localization imaging [28]. With advances in radiological techniques, this prevalence will likely decrease; however, occult ECS has often posed a clinical challenge in reported cases of RTH, as the thymus gland can serve as a primary site for ACTH-secreting neuroendocrine neoplasms. In the absence of evidence of a primary tumor, the recurrence of RTH after bilateral adrenalectomy prompted thymectomy due to suspicion of a primary neuroendocrine thymic tumor. In Cushing syndrome-affected patients, an increase in morbidity and mortality has been described, even after long-term hypercortisolism remission. For this reason, the need to perform major surgical interventions, like thymectomy, should be carefully considered [29-31].

## CONCLUSION

In the presented case, we have described the occurrence of RTH 8 months after recovery from ECS. In case of clinical suspicion, MR may be useful to characterize RTH in a non-invasive way. Increased awareness of this benign phenomenon and proper use of MRI chemical-shift imaging can prevent invasive diagnostic procedures in post-hypercortisolism patients.

## STRENGTHS AND LIMITATIONS

This article has described only one patient with RTH; to make more precise conclusions on MRI usefulness in this setting, a wider cohort of patients should be studied. However, the strength of this article is that it has included a review of all the available literature on RTH case reports.

## AUTHORS’ CONTRIBUTIONS

The authors confirm their contribution to this paper as follows; AM and VM wrote the initial draft of the article and prepared the final manuscript. EC, AP, PT, and MC contributed to the clinical care of the patient. LP and SC provided expert opinion on the results. All authors revised the manuscript and accepted the final version.

## Figures and Tables

**Fig. (1):**
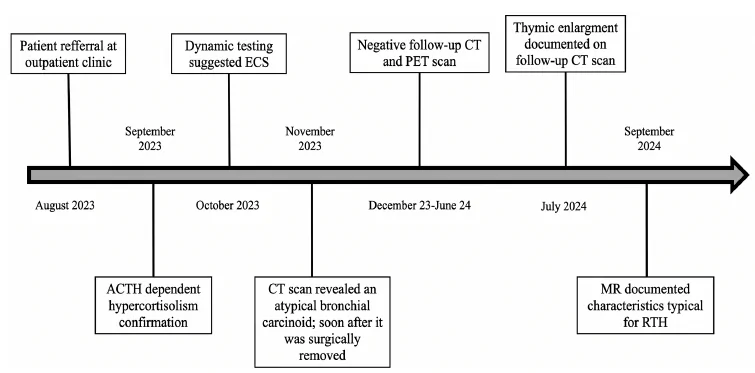
Patient’s history timeline. **Abbreviations:** ECS: Ectopic Cushing syndrome; RTH: Rebound thymic hyperplasia.

**Fig. (2):**
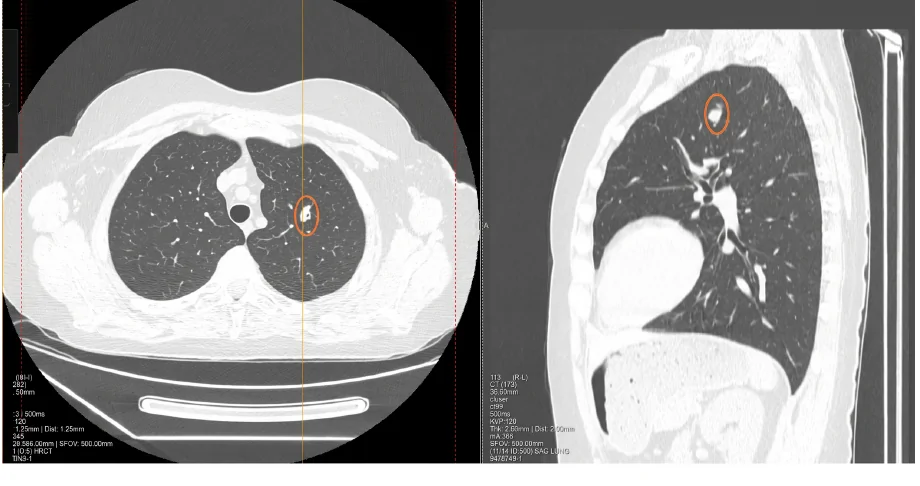
Ovalar peribronchial nodule of 14 mm of the upper-dorsal segment of upper left lobe. At histological examination, an atypical bronchial carcinoid was diagnosed.

**Fig. (3):**
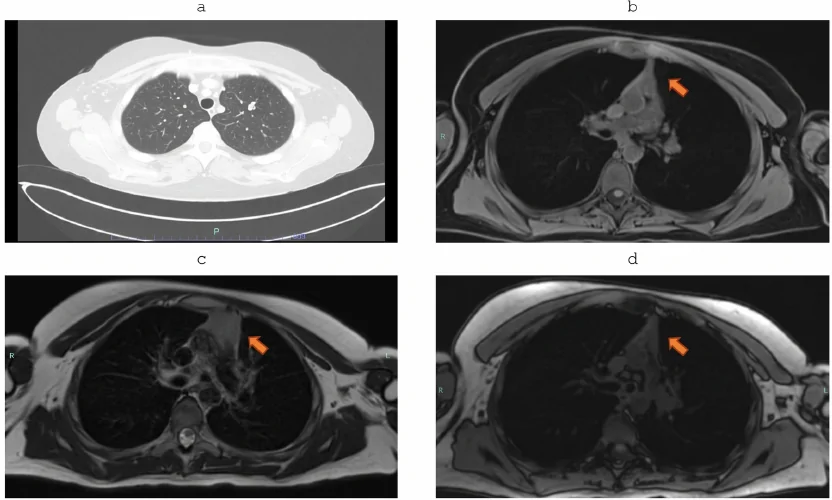
Anterior mediastinal enlargement at CT scan (**a**), which proved out to be RTH on MR and hyperintense in T2-weighted images (**b**), characterized by decreased signal intensity in out-of-phase images (**c, d**).

**Table 1:** Blood results at referral before and after EP pill (estrogen-progestin contraceptive pill) withdrawal.

-	**Value**	**Reference Range**
**Cortisol (nmol/l)**	1111.7	102.1-535.2
**DHEA-S (nmol/l)**	26.7	2.6-13.9
**Testosterone (nmol/l)**	4.26	0.24-2.74
**Delta-4-androstenedione (nmol/l)**	27.5	1.4-11.87
**17OH-progesterone (nmol/l)**	5.11	0.6-6.1
**TSH (mU/l)**	0.26	0.4-4
**FT3 (pmol/l)**	10.4	9-19.05
**FT4 (pmol/l)**	25.2	24.27-56.9
**Prolactin (mcg/l)**	14.7	2-29
**After the EP Pill Withdrawal**		
**Cortisol (nmol/l)**	3169.95	102.1-535.2
**LH (mU/l)**	<0.1	2.4-12.6
**FSH (mU/l)**	0.4	3.5-12.5
**Testosterone (nmol/l)**	3.29	0.24-2.74
**White blood cells (10^6^/m**cl)	13.55	4-10
**Neutrophil/lymphocytes (%)**	87/11	40-80/20-40
**Potassium (mmol/l)**	2.9	3.5-5
**Urinary free cortisol (nmol/24h)**	5026.9	46.9-375.22
**ACTH (pmol/l)**	14.97	1.1-11.01

**Abbreviations:** DHEA-S: Dehydroepiandrosterone sulfate; TSH: Thyroid-stimulating hormone; FSH: Follicle-stimulating hormone; LH: Luteinizing hormone; ACTH: Adrenocorticotropic hormone.

**Table 2:** Dynamic testing presumptive for ectopic Cushing syndrome.

-	**Value**	**Reference Range**
**UFC (nmol/24 hours)**	1086.49	46.9-375.2
**LNSC 1^st^**-**2^nd^-3^rd^** sp**e**c**imen (nmol/l)**	21.5-14.34-19.58	<9.66
**CRH Stimulation Test**		
**ACTH (pmol/l) 17.83 → 17.3 → 19.33; Δ 11.8%**		
**Cortisol (nmol/l) 548.96 → 510.34 → 529.65; Δ 3.7%**		
**Desmopressin stimulation test**		
**ACTH (pmol/l) 12.99 → 13.38 → 17.35; Δ 29.6%**		
**Cortisol (nmol/l) 474.48 → 419.31 → 455.17; Δ 8.6%**		
**High dexametasone doses suppression test**		
**Basal cortisol (nmol/l) 419.3 → 118.62 after suppression Δ 72%**		

** Abbreviations:** UFC: Urinary free cortisol; LNSC: Late-night salivary cortisol; CRH: Corticotropin-releasing hormone; ACTH: Adrenocorticotropic hormone.

**Table 3:** Post-operative examination showing hypercortisolism remission.

-	**Value**	**Reference Range**
Corsitol (nmol/l)	0.5	3.7-19.4
ACTH (pmol/l)	4.4	5-50
Potassium (mmol/l)	4	3.5-5
FSH/LH (mU/l)	1.3/1.7	3.5-12.5/2.4-12.6
17b-estradiol (pmol/l)	216	15-350
Testosterone (nmol/l)	<0.09	0.2-2.86
Delta-4-androstenedione (nmol/l)	<0.24	0.2-3.1
DHEA-S (nmol/l)	0.16	0.35-4.3
17OH-progesterone (nmol/l)	0.32	1.97-5.78
White blood cells (10^6^/mcl)	7.8	4-10
Neutrophil/lymphocytes (%)	65.4/24.7	40-80/20-40

**Abbreviations:** ACTH: Adrenocorticotropic hormone; FSH: Follicle-stimulating hormone; LH: Luteinizing hormone; DHEA-S: Dehydroepiandrosterone sulfate.

**Table 4:** A review of currently reported RTH cases.

**Gender**	**Age**	**Hypercortisolism Etiology**	**Month of RTH Presentation After Hypercortisolism Resolution**	**Month of Spontaneous Remission/surgical Intervention**	**Author**
**M**	15	CD	3	ND	Doppman *et al.* [20]
**F**	27	ECS bronchial carcinoid	6	ND	Doppman *et al.* [20]
**M**	35	ECS occult origin	3	-	Doppman *et al.* [20]
**F**	59	ECS bronchial carcinoid	8	Thymectomy	Tabarin *et al.* [9]
**M**	44	ECS bronchial carcinoid	5	Thymectomy	Tabarin *et al.* [9]
**M**	30	ECS occult origin	11	Thymectomy	Tabarin *et al.* [9]
**F**	33	ECS bronchial carcinoid	8	Thymectomy	Tabarin *et al.* [9]
**F**	37	ECS bronchial NE carcinoma	11	12 months	Tabarin *et al.* [9]
**F**	48	CD	6	Thymectomy	Schmidt *et al.* [18]
**M**	33	ECS occult origin	6	Thymectomy	Schmidt *et al.* [18]
**F**	42	ND	Mean 16 months	ND	Hanson *et al.* [25]
**ND**	ND	ND	Mean 16 months	ND	Hanson *et al.* [25]
**ND**	ND	ND	Mean 16 months	ND	Hanson *et al.* [25]
**ND**	ND	ND	Mean 16 months	ND	Hanson *et al.* [25]
**F**	48	ECS occult origin	8	38 months	Neto *et al.* [19]
**M**	27	ECS bronchial carcinoid	3	Thymectomy	Icard *et al.* [21]
**F**	23	Adrenal cancer	8	36 months	Delitala *et al.* [22]
**F**	44	CD	ND	Thymectomy	Rivero *et al.* [23]
**F**	5 months	Adrenal adenoma	6	FNA benign histology, remission at 12 months	Hwang *et al.* [24]

**Abbreviations:** CD: Cushing disease; ECS: Ectopic Cushing syndrome.

## Data Availability

The data will be made available by the corresponding author upon reasonable request.

## LIST OF ABBREVIATIONS

RTH: Rebound Thymic Hyperplasia
ECS: Ectopic Cushing syndrome
CS: Cushing Syndrome
ACTH: Adrenocorticotropic Hormone
UFC: Urinary Free Cortisol
LNSC: Late Night Salivary Cortisol
DHEA-S: Dehydroepiandrosterone Sulfate
TSH: Thyroid-stimulating Hormone
FSH: Follicle-stimulating Hormone
LH: Luteinizing Hormone
VATS: Video-assisted Thoracic Surgery
18FDG PET-CT: 18-fluorodeoxyglucose PET–CT
MR: Magnetic Resonance

## STANDARDS OF REPORTING

CARE guidelines were followed.
